# Cooperation between Strain-Specific and Broadly Neutralizing Responses Limited Viral Escape and Prolonged the Exposure of the Broadly Neutralizing Epitope

**DOI:** 10.1128/JVI.00828-17

**Published:** 2017-08-24

**Authors:** Colin Anthony, Talita York, Valerie Bekker, David Matten, Philippe Selhorst, Roux-Cil Ferreria, Nigel J. Garrett, Salim S. Abdool Karim, Lynn Morris, Natasha T. Wood, Penny L. Moore, Carolyn Williamson

**Affiliations:** a Institute of Infectious Disease and Molecular Medicine and Division of Medical Virology, Department of Pathology, Faculty of Health Sciences, University of Cape Town, Cape Town, South Africa; b Centre for HIV and STIs, National Institute for Communicable Diseases (NICD) of the National Health Laboratory Service, Johannesburg, South Africa; c Division of Computational Biology, Department of Integrative Biomedical Sciences, Faculty of Health Sciences, University of Cape Town, Cape Town, South Africa; d CAPRISA, University of KwaZulu-Natal, Durban, South Africa; e Discipline of Public Health Medicine, School of Nursing and Public Health, University of KwaZulu-Natal, Durban, South Africa; f Department of Epidemiology, Columbia University, New York, New York, USA; g Faculty of Health Sciences, University of the Witwatersrand, Johannesburg, South Africa; h National Health Laboratory Service, Johannesburg, South Africa; Emory University

**Keywords:** broadly neutralizing antibodies, deep sequencing, glycan holes, viral escape, glycan shield, V3-glycan supersite, neutralization escape, N332 glycan, helper/cooperating NAb responses

## Abstract

V3-glycan-targeting broadly neutralizing antibodies (bNAbs) are a focus of HIV-1 vaccine development. Understanding the viral dynamics that stimulate the development of these antibodies can provide insights for immunogen design. We used a deep-sequencing approach, together with neutralization phenotyping, to investigate the rate and complexity of escape from V3-glycan-directed bNAbs compared to overlapping early strain-specific neutralizing antibody (ssNAb) responses to the V3/C3 region in donor CAP177. Escape from the ssNAb response occurred rapidly via an N334-to-N332 glycan switch, which took just 7.5 weeks to reach >50% frequency. In contrast, escape from the bNAbs was mediated via multiple pathways and took longer, with escape first occurring through an increase in V1 loop length, which took 46 weeks to reach 50% frequency, followed by an N332-to-N334 reversion, which took 66 weeks. Importantly, bNAb escape was incomplete, with contemporaneous neutralization observed up to 3 years postinfection. Both the ssNAb response and the bNAb response were modulated by the presence/absence of the N332 glycan, indicating an overlap between the two epitopes. Thus, selective pressure by ssNAbs to maintain the N332 glycan may have constrained the bNAb escape pathway. This slower and incomplete viral escape resulted in prolonged exposure of the bNAb epitope, which may in turn have aided the maturation of the bNAb lineage.

**IMPORTANCE** The development of an HIV-1 vaccine is of paramount importance, and broadly neutralizing antibodies are likely to be a key component of a protective vaccine. The V3-glycan-targeting bNAb responses are among the most promising vaccine targets, as they are commonly elicited during infection. Understanding the interplay between viral evolution and the development of these antibodies provides insights that may guide immunogen design. Our work contrasted the dynamics of the early strain-specific antibodies and the later broadly neutralizing responses to a common Env target (V3C3), showing slower and more complex escape from bNAbs. Constrained bNAb escape, together with evidence of contemporaneous autologous virus neutralization, supports the proposal that prolonged exposure of the bNAb epitope enabled the maturation of the bNAb lineage.

## INTRODUCTION

While antiretroviral therapy (ART) is highly effective at controlling HIV-1 and significant advances have been made in using ART treatment for prevention, an effective vaccine still represents the greatest chance of ending the HIV/AIDS pandemic. Broadly neutralizing antibodies (bNAbs) are one of the main focus points for HIV-1 vaccine research due to their ability to block infection by different subtypes and strains of HIV-1. Although the elucidation of B cell evolutionary pathways to bNAb development has provided a promising approach to immunogen design, these studies have not yet translated into bNAb-eliciting vaccines. Immunogen design strategies can be augmented by studies of natural infection that elucidate viral evolution in response to early strain-specific and later bNAb responses.

There are six known sites on the viral envelope targeted by bNAbs, with the ontogeny of only three of these bNAb specificities characterized to date: the CD4 binding site, V1V2, and V3-glycan (1–6). Of these, the V3-glycan targeting bNAb responses may be easiest to elicit via vaccination, because they are among the most common and potent bNAb responses in infected individuals (7–10), can develop relatively early in infection, and do not always require extensive somatic hypermutation (4, 11, 12). Furthermore, these bNAbs have the highest expected therapeutic effectiveness (10) and indeed have been shown to suppress viremia in passive immunization studies (13). Therefore, considerable interest exists in the field with regard to understanding the virus-antibody dynamics relevant to the development of, and escape from, this class of bNAbs.

Glycans on the surface of gp120 are important modulators of antibody neutralization, with some of the most potent bNAbs requiring glycan contacts (8, 12, 14–19). Most V3-glycan bNAbs are highly dependent on the N332 glycan (9, 12, 19–21), although this varies within bNAb lineages (6, 21, 22), and loss of this glycan has been associated with viral escape (6, 13, 16, 20, 21, 23). V3-glycan bNAbs also target the ^324^GDIR^327^ motif at the base of the V3 loop, and escape mutations in this motif, particularly at positions D325 and R327, have been shown to reduce neutralization (24, 25). In addition, an increase in V1 loop length, and its glycosylation content, can mediate escape from this class of bNAbs (5, 6, 24, 26, 27).

Strain-specific neutralizing antibody (ssNAb) responses, which occur in early infection, drive viral evolution and thus are responsible for molding the virus that elicits the bNAb response later in infection. The interplay between early ssNAb responses and the later bNAb responses has not received significant attention, with only a few studies conducted to date (23, 28–30). A study of two individuals who developed V3-glycan-targeting bNAb responses, one of which is the subject of this paper, found that escape from ssNAb responses resulted in creation of the bNAb epitope (16). Such studies provide important insights into the role of ssNAb responses in creating a favorable environment for the development of bNAbs (30).

In order to evaluate virus-antibody dynamics relevant to the V3-glycan class of bNAbs, we studied an HIV-1-infected individual, CAP177, who developed an ssNAb response to the V3/C3 region at 19 weeks postinfection (wpi) (31), as well as a V3-glycan bNAb response, which first emerged at approximately 1 year postinfection and matured gradually to reach 52% breadth by 3 years postinfection (16, 32). Using deep sequencing coupled with neutralization phenotyping, we showed rapid escape from ssNAb responses but slow, incomplete escape from bNAbs. These data indicate that selective pressure exerted by the ssNAb response have constrained bNAb escape, thereby prolonging exposure of the bNAb epitope and potentially aiding the maturation of the bNAb response.

## RESULTS

Sequences were generated from a total of 25 plasma samples from donor CAP177, collected at two weekly intervals for the first 3 months, monthly up to the end of the first year, and quarterly until 4 years postinfection. Using next-generation sequencing and the primer ID method (33), we generated a total of 92,319 consensus sequences covering the V1V2 (255 bp) and V3/C3 (333 bp) regions, with an average of 1,846 (339 to 12,840) consensus sequences generated per time point for each region.

### Viral population dynamics associated with early neutralizing antibody pressure.

**FIG 1:**
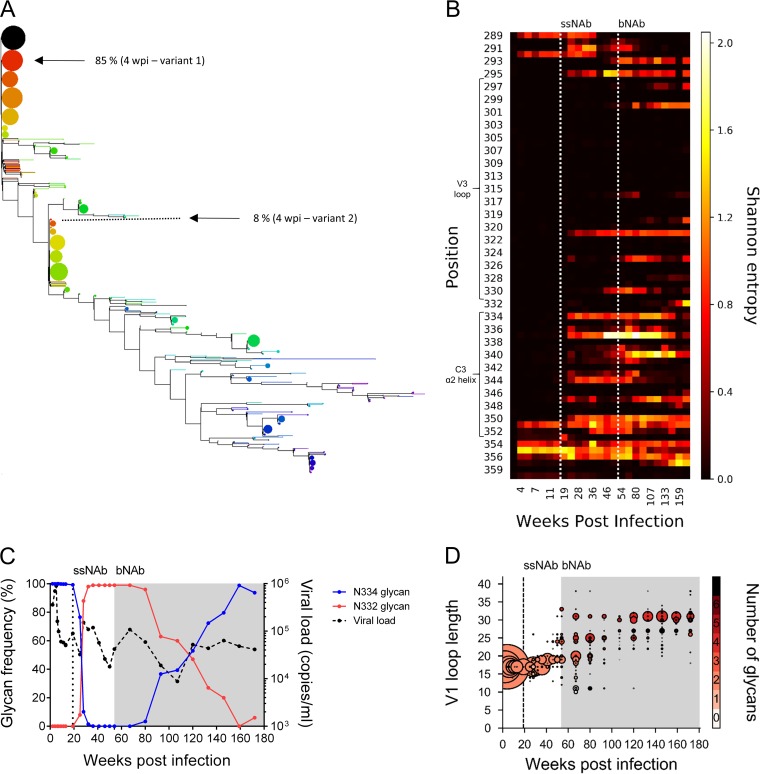
Dynamics of gross changes in the V1 and V3/C3 regions of CAP177 *env*. (A) Phylogeny of the V3/C3 regions, generated using the maximum likelihood approach. Colors indicate time from 2 wpi (red) to 172 wpi (purple). Multiple-variant transmission is indicated (arrows), with one major variant detected at 2 wpi and two distinct variants identified at 4 wpi. Bubble sizes are proportional to the frequency of the given sequence. (B) Heatmap showing positional entropy over *env* V3C3 regions. Time points corresponding to the emergence of ssNAb and bNAb responses are indicated (dotted lines). (C) The frequencies of glycosylation at positions N334 (blue) and N332 (red) are indicated on the left *y* axis, with changes in viral load (black) shown on the right *y* axis. (D) Changes in V1 loop length and glycosylation content over time. Bubbles indicate the proportion of viruses with a given V1 loop length (*y* axis) and number of glycan sequons (color). Bubble sizes were normalized for sequencing depth and scaled by viral load. The emergence of ssNAbs at 19 wpi is indicated by a dotted line, with time points corresponding to the emergence of the breadth response shaded gray.

Sequences from the first HIV-1-positive plasma sample (HIV PCR positive, antibody negative; estimated to be 2 wpi) were highly homogenous: 98% of the sequences were identical, with the remaining sequences differing by dispersed single-nucleotide changes. A distinct, closely related variant which grouped separately on a maximum likelihood tree (Fig. 1A) was detected at 4 wpi (frequency of 8.3%). This variant differed from the transmitted/founder virus by 6.5% in the V1V2 region and 1.2% in the V3C3 region, indicating a multivariant transmission event (34). Given the depth of sampling at 2 wpi, we had a 95% probability of detecting a variant present at a >0.25% frequency, suggesting either that the minor variant was present at extremely low levels at 2 wpi or that its migration from the local site of infection was delayed.

Sequence variation over time was evaluated by plotting the Shannon entropy for each position (Fig. 1B). Prior to the first detectable ssNAb response at 19 weeks, there was no increase in entropy in the V3 region. However, an increase in entropy was observed in C3 (positions 351 to 360) at 4 wpi, which may have been due to immune pressure from nonneutralizing antibody effector functions or cytotoxic T-lymphocytes, as this change occurred in a known cytotoxic T lymphocyte epitope (35), restricted by the participant's human leukocyte antigen allele (Cw*0401).

From 19 weeks, concurrent with the emergence of ssNAbs, there was elevated entropy at position 295, at position 321 in V3, and at various positions in the C3 α2-helix (334, 337, 339 to 344, 347, and 350). We have previously reported that escape from the early ssNAb response was mediated by a shift of the N334 glycan to position N332 (16). Both the major variant, detected at 2 wpi, and the minor variant, detected at 4 wpi, had the N334 glycan. After the development of ssNAb responses, there was a rapid shift from 100% of variants harboring the N334 glycan to a population dominated by N332 glycan viruses (Fig. 1C). The frequency of the N334 glycan variant fell to 0.13% by 36 wpi and was undetectable by 54 wpi (95% probability to detect variant at a frequency of 0.14%). These data suggest that the early ssNAb made contacts within the α2-helix of C3 and the N334 glycan.

### Early C3 ssNAb and V3-glycan bNAb epitopes were partially overlapping.

The bNAb response in CAP177 belongs to the V3-glycan class, with dependence on the N332 glycan (16). A number of V3-glycan bNAbs target the ^324^GDIR^327^ motif and are influenced by changes in the V1 loop (5, 6, 24, 26, 27), as well as being largely dependent on glycosylation at N332 and N301 (12, 24).

We assessed viral evolution after the emergence of bNAb and compared sites under immune pressure with those targeted by the ssNAbs described above to determine whether these epitopes overlapped. Concurrent with the emergence of the bNAb response at ∼54 wpi, several positions increased in sequence entropy (sites 300, 325, 330, 337, and 340), which continued to increase as the bNAb response matured. The V1 loop underwent a pronounced expansion in length and glycan number (Fig. 1D) between 41 and 46 wpi (6 to 13 weeks prior to detection of cross-neutralization). Large insertions in the V1 loop of 7 to 20 amino acids (aa) emerged at 54 wpi, with the greatest variation in loop length seen over 54 to 73 weeks, where the V1 loop length varied from 10 to 37 aa. While some V1 loop deletion variants were present at 80 wpi, the overwhelming trend was toward longer V1 loops, with this increase in loop length associated with a larger number of glycosylation sites in the V1 loop. Reversion of N332 to the N334 glycan occurred later, first detected at 80 wpi (3.39% frequency), and reached 52.9% frequency at 120 wpi when the bNAb response peaked.

**FIG 2:**
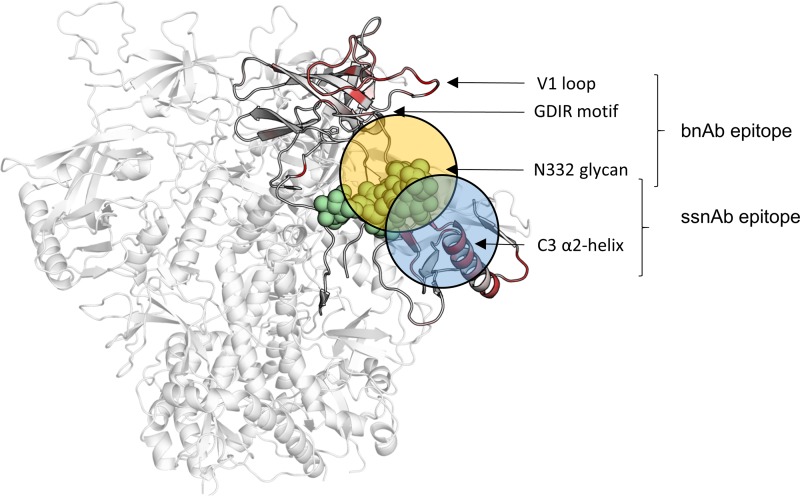
Predicted epitopes for the early ssNAb and bNAb responses. A trimer model of CAP177 gp160 (white), with sequence entropy mapped onto the structure, for the regions covered by the NGS data (light gray), highlighted using a white-to-red gradient. Predictions of the approximated epitopes for the ssNAb (blue circle) and bNAb (orange circle) responses are indicated. Glycosylation at position N332 is shown (green spheres).

Both ssNAb and bNAb responses were influenced by the presence/absence of the N332/N334 glycan, suggesting that these two responses target partially overlapping epitopes. Based on the timing of changes in sequence entropy, the predicted ssNAb epitope was shifted toward the C3 α2-helix, while the putative bNAb epitope was located further toward the V3 loop (Fig. 2).

### Escape from the bNAb response includes, but is not limited to, loss of the N332 glycan.

We explored whether the loss of the N332 glycan conferred escape from the bNAb response in this individual, as seen with other V3-glycan bNAbs (12, 13, 16, 36). To investigate this, we generated six functional *envelope* (*env*) clones from 107 and 120 wpi. These time points corresponded to the time of peak breadth, and viruses harboring either the N332 or N334 glycan were present in roughly equal proportions. We evaluated the change in neutralization sensitivity if the N332 glycan was replaced by an N334 glycan or vice versa, depending on the wild-type (WT) glycosylation status of each of the 6 Env proteins.

**FIG 3:**
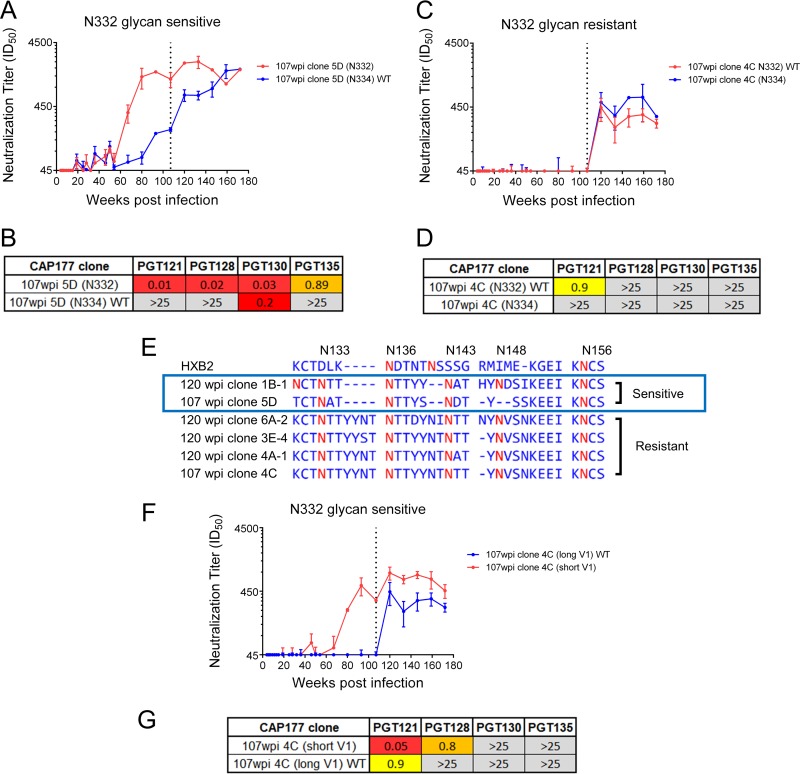
Effect of an N332-to-N334 glycan shift and V1 loop length on escape from bNAb activity. (A) CAP177 plasma neutralization titers (ID_50_) for N332 (red) and N334 (blue) glycan variants of CAP177 clone 5D. (B) Sensitivity of the N332 and N334 glycan variants to a panel of V3-glycan supersite MAbs (IC_50_, in μg/ml). (C) CAP177 plasma neutralization titers (ID_50_) for N332 (red) and N334 (blue) glycan variants of CAP177 clone 4C. (D) Sensitivity of the N332 and N334 glycan variants to a panel of V3-glycan supersite MAbs (IC_50_, in μg/ml). (E) Sequence alignment for the V1 loop of several clones, with key V1 loop glycans indicated (red). Clones with either sensitive or resistant neutralization phenotypes are indicated, with those from the resistant group having V1 loops which were between 5 and 9 aa longer. (F) CAP177 plasma neutralization titers (ID_50_) to a resistant clone (clone 4C) from 107 wpi (blue), as well as to a variant of this clone with a shorter V1 loop (red) taken from a sensitive clone (clone 5D). The dotted line indicates the plasma sampling point (107 wpi) from which the clone was isolated. (G) Sensitivity of V1 loop length variants to a panel of V3-glycan supersite MAbs (IC_50_, in μg/ml). The dotted line indicates the plasma sampling point (107 wpi) from which the clones were isolated.

For two 107-wpi clones (5D and 1B-1), a switch from the N332 to N334 glycan corresponded with a decrease in sensitivity to CAP177 plasma (Fig. 3A and data not shown). Reversion of this glycan (N332 to N334) similarly resulted in a decreased sensitivity to a panel of four V3-glycan monoclonal antibodies (MAbs) (Fig. 3B), suggesting that the selection of the N334 glycan in these clones was driven by the bNAb response. Of note, both of these clones showed contemporaneous neutralization (neutralization of virus by plasma from the same time point) and precontemporaneous neutralization (neutralization of virus by plasma from time points prior to the isolation of the virus), with titers of >1:100, regardless of whether they had N332 or N334 glycans, suggesting incomplete escape.

In contrast, four 107 wpi clones (4C, 4A-1, 6A-2, and 3E-4) showed no difference in CAP177 plasma neutralization sensitivity between the N332 (WT) and N334 glycan mutant (Fig. 3C and data not shown). Furthermore, unlike clones 5D and 1B-1, we observed no contemporaneous neutralization for clones 4C, 4A-1, 6A-2, and 3E-4.

In addition, these mutants displayed greater resistance to the panel of V3-glycan MAbs than clone 5C at 107 wpi and clone 1B at 120 wpi (Fig. 3D and data not shown), regardless of N332 glycan status. This suggested N332/N334 glycan-independent escape in these clones.

A possible N332-independent escape pathway for V3-glycan supersite bNAbs is through point mutations in the conserved ^324^GDIR^327^ motif, which is a known contact point for many V3-glycan bNAbs (12, 24, 25). Although we observed a transient change in the GDIR motif after the development of breadth, this involved a D325N mutation which only reached a peak of ∼20% frequency (by 80 wpi), after which this mutation reverted to the WT 325D (98% frequency by 133 wpi) (data not shown). This mutation is not associated with PGT121/128-like bNAb escape (8, 13, 24, 25, 36), and given its transient nature, the D325N mutation is unlikely to have contributed significantly to bNAb escape in CAP177.

### Changes in V1 loop length mediated escape from V3-glycan bNAb responses.

Longer V1 loops, with more glycans, can reduce neutralization by V3-glycan bNAbs (24, 26, 27). Given that there was a pronounced expansion of the V1 loop associated with the development of bNAbs (Fig. 1D), we assessed whether this region contributed to escape in CAP177. We found that clones where the N332/N334 glycan switch did not affect neutralization sensitivity (4C, 4A-1, 6A-2, and 3E-4) had V1 loops which were, on average, seven amino acids longer (31 to 32 aa long) than those of clones which were sensitive to the N332/N334 glycan switch (23 to 26 aa) (Fig. 3E). These clones all contained a 4-aa insertion upstream of N136 and a 2-aa insertion before N143. Additionally, these clones had a 2-aa insertion after Y147 relative to clone 5D, introducing a new glycan site at N148, although this insertion was also seen in the sensitive clone 1B-1. The sensitive clone 1B-1 and the resistant clone 6A-2 had a further 1-aa insertion prior to Y147. We therefore constructed a series of chimeric envelopes, swapping the longer V1 loop from the resistant clone 4C with the shorter loop from the sensitive clone 5D. This change to the V1 loop increased neutralization sensitivity, with the shorter V1 loop variant of clone 4C also becoming sensitive to neutralization by contemporaneous CAP177 plasma (Fig. 3F). The V1 loop swap mutants also showed increased sensitivity to V3-glycan MAbs PGT121 and PGT128 (Fig. 3G), confirming that the increased V1 loop length, and possibly the increased glycan content or the modified glycan positions of the V1 loop, was an alternative escape pathway from the V3-glycan bNAb response.

When we carried out the reciprocal experiment, introducing the longer V1 loop from the neutralization-resistant clone 4C into neutralization-sensitive clone 5D (with the N332 glycan), we saw no change in the neutralization sensitivity (data not shown). This may be due to sequence variation in regions adjacent to the V1 loop, which may have influenced the conformation of the loop such that it was stabilized or positioned away from the V3 bNAb epitope.

### Longer V1 loops block access to the V3-glycan supersite.

**FIG 4:**
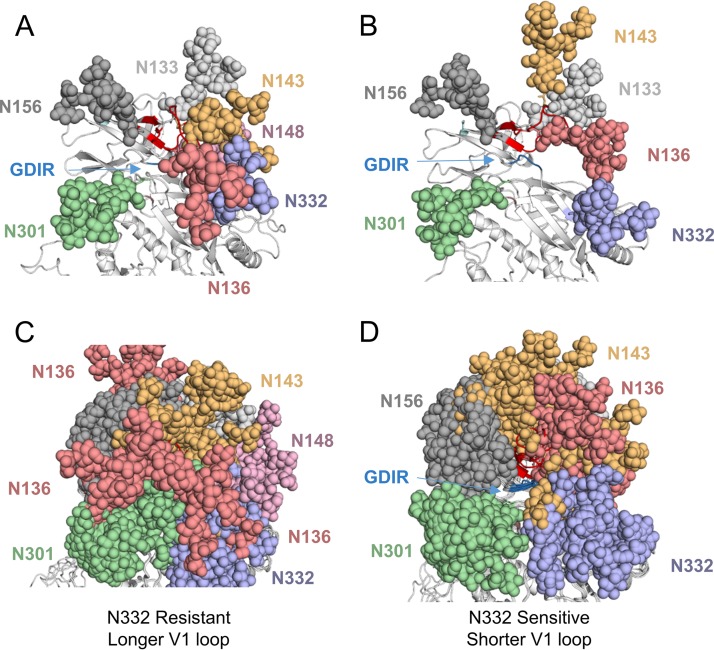
Effect of V1 loop length and glycosylation on V3-glycan supersite accessibility. Modeled trimeric structures of CAP177 neutralization-resistant (A) and -sensitive (B) clones (cartoon representation), with Man-9 glycans (colored spheres) attached to potential N-linked glycan sites (with clash resolution). The key V3-glycan bNAb ^324^GDIR^327^ motif is indicated (dark blue). An overlay of several modeled trimeric structures of CAP177 neutralization-resistant (C) and -sensitive (D) clones (cartoon representation) is shown.

To assess the mechanism by which V1 loop length affected the V3-glycan bNAb supersite, we used the sequences of the sensitive and resistant clones, 5D and 4C, respectively, to generate modeled structures for these variants. Both variants had N332 glycan; however, 4C had a longer V1 loop (31 aa) than clone 5D (24 aa). Man-9 glycans were added to potential N-linked glycosylation sites *in silico* to produce near-fully glycosylated gp160 trimer models for each clone. In this model, we found that the V1 loop was orientated in such a way that the glycan at position N136, which was largely conserved over approximately the first 3 years of infection (data not shown), could potentially interfere with bNAb-N332 glycan interactions, bNAb-V3 backbone interactions (including the GDIR motif), or glycan-glycan interactions involving N332 (Fig. 4A). In contrast, in the modeled structure for the neutralization-sensitive variant (clone 5D), the V1 loop and its associated glycans were orientated in such a way that they did not interfere with access to the GDIR motif or to disrupt bNAb-N332 glycan interactions (Fig. 4B).

Given that the V1 loop could be positioned in various orientations by the modeling algorithm, we repeated the modeling and glycosylation steps 10 times for each clone. The iterative modeling provided insights into the potential range of motion of the different V1 loops and their associated glycans. Overlaying the replicate models for the resistant clone 4C highlighted the wide range of V1 loop conformations, with the associated V1 glycans positioned in a number of different orientations, particularly the glycan at position N136 (Fig. 4C), with the GDIR motif and N332 glycan occluded. Overlaying the models from the sensitive clone 5D, however, showed a much lower range of motion for this V1 loop and its associated glycans, with the GDIR motif and N332 glycan exposed. Consequently, almost all of the V1 glycans displayed relatively tight clustering (Fig. 4D). Furthermore, the sensitive clone with a shorter V1 loop had a noticeable hole in the glycan shield over the conserved GDIR motif, which was not exposed in the resistant clone with the longer V1 loop.

### No difference in infectivity of N332 and N334 glycan clones.

**FIG 5:**
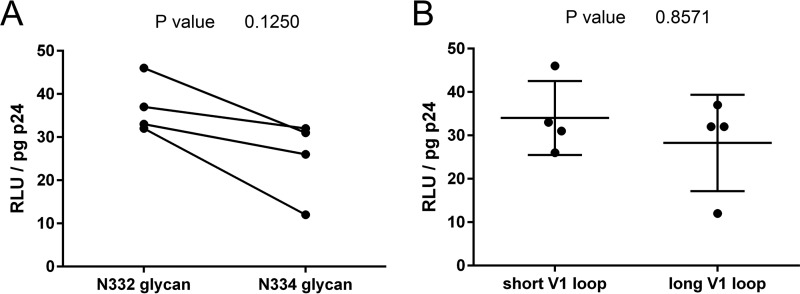
Effect of N332/N334 glycosylation and V1 loop length on viral entry efficiency. (A) Differences in entry efficiency for N332 glycan and matched N334 glycan IECs are shown, with the Wilcoxon matched-pairs test *P* value indicated. (B) Differences in entry efficiency for unmatched IECs with long (31 or 32 amino acids) or short (23 or 26 amino acids) V1 loops are shown, with the Mann-Whitney test *P* value indicated.

The N334 glycan is found in approximately 20% of global viruses, with the N332 glycan occurring in ∼70% of viruses (21, 36). Although N334 viruses are clearly replication competent, the lower global frequency of N334 suggests this form is less favorable. To evaluate if the N334 glycan was associated with lower viral infectivity, we generated four pairs of matched infectious envelope clones (IECs) from clones 1B-1, 5D, 4C, and 3E4 (listed in Fig. 3E), such that each clone had an N332 and N334 glycan variant. Clones 6A-2 and 4A-1 did not produce IECs with sufficient infectivity; thus, they were excluded. The N334 glycan-containing version of each clone had moderately lower infectivity than the matched N332 clone, with a median fold difference of 0.73 (0.375 to 0.865) (Fig. 5A); however, this was not statistically significant (*P* = 0.1250 by Wilcoxon matched-pairs test). In addition, using the same set of IECs, we tested the effect of long and short V1 loops on viral infectivity (unmatched for V1 loop length), but we found no evidence that longer V1 loops affected entry efficiency (Fig. 5B) (*P* = 0.8571 by Mann-Whitney test).

### Escape from bNAb response was slower than that for ssNAbs.

We next compared the rates of escape from bNAbs, which target more conserved regions of the Env, to those of ssNAbs that are known drive rapid escape (31, 37–39). In CAP177, time to escape (the time point at which escape mutations were present at >50% frequency in viral sequence data) was measured from the first detectable ssNAb response at 19 wpi and the first detectable breadth at 54 wpi (neutralization of more than two heterologous viruses, with a 50% inhibitory dilution [ID_50_] of >45).

**FIG 6:**
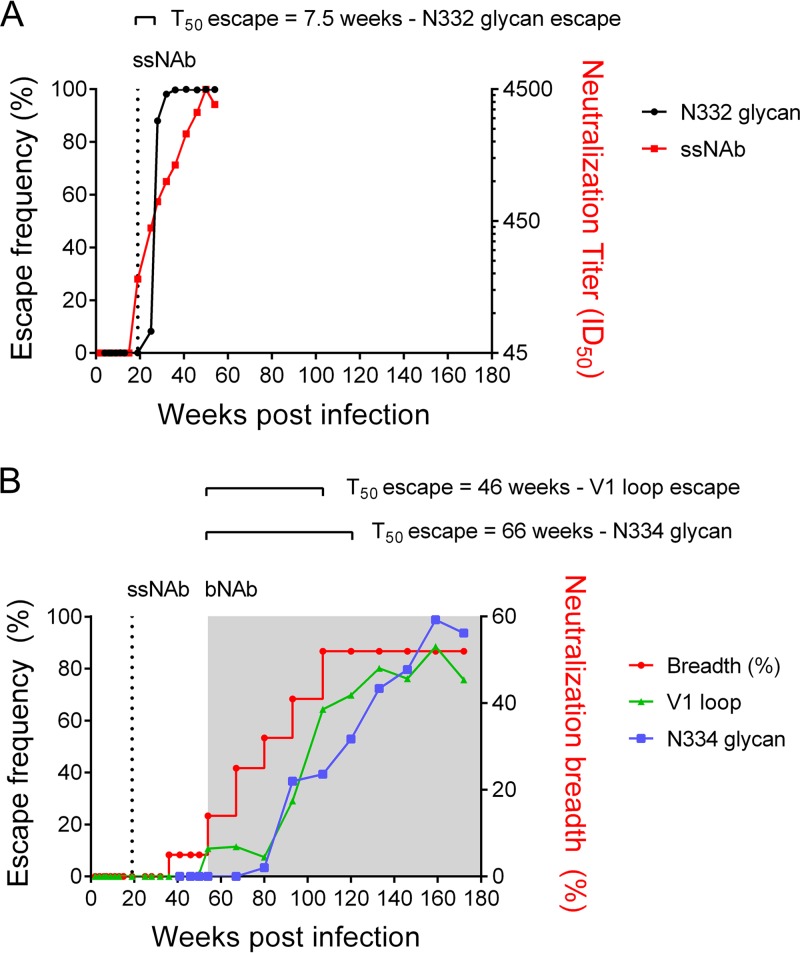
Kinetics of escape from ssNAb and bNAb responses. (A) Rate of escape from early ssNAb responses via the N334-to-N332 glycan shift (black) is shown, with 50% escape reached 11 weeks after the emergence of the ssNAb response (red). (B) Rates of escape from the bNAb response, including escape via the V1 loop (green), and reversion to the N334 glycan (blue). The incremental development of neutralization breadth is indicated (red).

The N334/N332 glycan shift was the major escape pathway from the early ssNAb response. This N334-to-N332 glycan shift occurred rapidly, taking just 7.5 weeks to reach 50% frequency and only 11 weeks to reach 90% replacement of the transmitted/founder sequence (Fig. 6A). In contrast, escape from the bNAb response was much slower. The first escape pathway was the lengthening of the V1 loop (V1 loop length of ≥31 aa), which took 46 weeks to reach 50% frequency and 105 weeks to reach a peak of 89% frequency. The second bNAb escape pathway was a reversion of the N332 glycan to N334, which took 66 weeks to reach 50% and 105 weeks to reach 90% frequency (Fig. 6B). Thus, escape from bNAbs took 40 weeks longer to achieve than escape from ssNAbs in this donor, despite the fact that we observed no significant fitness costs associated with the reversion to N334.

**FIG 7:**
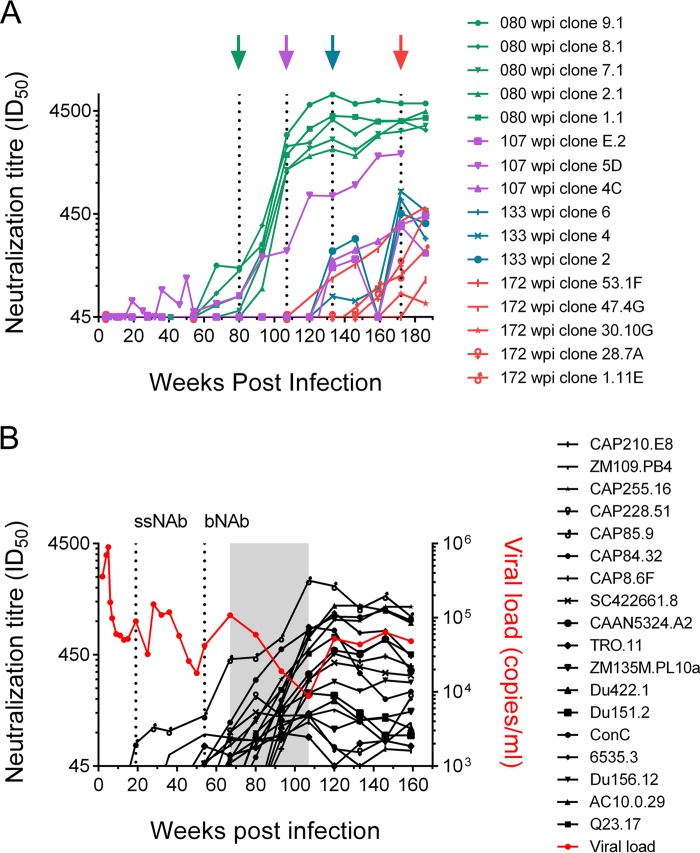
Contemporaneous neutralization and changes in viral load associated with the development of the bNAb response. (A) Longitudinal autologous neutralization ID_50_ titers over the development of bNAbs are depicted for several viral clones, sampled from 80 wpi (green), 107 wpi (purple), 133 wpi (blue), and 155 wpi (red), showing contemporaneous neutralization at all time points. (B) The increase in neutralization for longitudinal CAP177 plasma to a panel of 18 heterologous, cross-clade viruses (black lines) overlaps a 40-week period (gray shading) of a sustained, greater-than-1-log decrease in viral load (red). Time points corresponding to the emergence of ssNAb and bNAb responses are indicated (dotted line).

To evaluate whether slow escape was associated with contemporaneous neutralization, we tested three to five clones from 80, 107, 133, and 156 wpi against longitudinal plasma (Fig. 7A). We found contemporaneous neutralization for the majority of clones at each time point, with the exception of 107 wpi, where two of the three clones tested only became sensitive at the following time point (120 wpi). These results support the finding of slow escape from the bNAb response.

The presence of contemporaneous neutralization indicates incomplete escape from the antibody response and led us to evaluate whether antibodies played a role in transiently controlling viral replication. Thus, we investigated changes in viral load in relation to the development of heterologous neutralization (measured as plasma neutralization titers against a panel of 18 cross-clade viruses [32]). We observed a sustained drop in viral load of greater than 1 log from 67 wpi (just 13 weeks after the emergence of bNAbs) until the peak of the breadth responses at ∼107 wpi (Fig. 7B).

## DISCUSSION

The generation of bNAb responses through vaccination remains an essential question in HIV research. In infection, these antibodies generally take years to evolve and many are extensively hypermutated (4, 12, 40–44), suggesting a requirement for prolonged antigen exposure. Here, we investigated the interplay between ssNAbs and bNAbs in an individual where there was conflicting selection pressure on the N332/344 glycan. Shifting of the N332 glycan to N334 resulted in rapid escape from ssNAbs, while escape from bNAbs was slow and complex, involving multiple pathways.

Escape from NAbs can occur even in response to low antibody titers (45). As a result, circulating viruses usually are not neutralized by contemporaneous plasma (45–49). This rapid viral escape likely deprives B cells of antigenic stimulation, prematurely terminating their development. However, we observed contemporaneous neutralization by CAP177 plasma at several time points, suggesting incomplete viral escape. Examples of contemporaneous neutralization have been observed before (28, 48–50), including in another bNAb donor, CAP256, who developed a potent V2 response. The slow escape seen here and in other bNAb donors (3, 28, 30), together with contemporaneous autologous virus neutralization, supports the notion that bNAb development requires prolonged exposure to the epitope.

One explanation for the slow reversion to N334 is a possible difference in infectivity between N332 and N334 glycan viruses. The higher global prevalence of the N332 glycan relative to N334 suggests that this glycan provides a fitness advantage to the virus. However, while we found that viruses with N334 had slightly lower infectivity than matched N332 viruses, this was not significant, indicating that the relative entry efficiency of viruses with N332 or N334 glycans was not responsible for the low rate of escape in this case. Additionally, it is clear that N334 viruses are replication competent and can be selected for *in vivo*, as shown previously in V3-glycan bNAb donors CAP177 and CAP314 (16).

Alternatively, the slow escape from the bNAbs in CAP177 is a consequence of competing selection pressures mediated by the ssNAb response, which targets the N334 glycan. In the context of passive immunization, escape from V3-glycan bNAbs via the removal of the N332 glycan can occur rapidly. In the recent V3-glycan bNAb passive infusion trial, over 90% of viruses had escaped by elimination of the N332 glycan within 4 weeks of infusion of the 10-1074 V3-glycan bNAb (13). This is in striking contrast to the slow escape in CAP177 and suggests that the ssNAb response constrained V3-glycan bNAb escape pathways, resulting in the observed slow and incomplete escape. The concept that ssNAbs can facilitate bNAb development was originally demonstrated by Moore et al., who first described the role of viral escape in the development of breadth (16). The cooperation between ssNAbs and bNAbs was elucidated in more detail by Gao et al., who isolated two antibody lineages and showed that escape from one lineage created a viral variant that was recognized by the second CD4 binding site bNAb lineage (30). Overall, these studies emphasize the importance of ssNAb response in shaping bNAb development.

Interestingly, the increase in V1 loop length was a more complete escape pathway from polyclonal responses than that of N334, evidenced by the fact that the N334 escape variants were still susceptible to contemporaneous neutralization, which was not seen for the V1 loop escape variants. This is supported by two recent studies (26, 27) which showed that changes in the V1 loop were primarily responsible for V3-glycan bNAb escape, with no escape mutations detected in the GDIR motif or in the N332 glycan site. Additionally, a number of studies have implicated changes in V1 loop glycosylation, particularly position N136/137, as being important for many V3-glycan bNAbs (5, 21, 25–27, 51). We found that the N136 glycan was largely conserved throughout infection. However, our molecular modeling results indicate that escape was likely the result of this glycan changing its relative orientation as the V1 loop lengthened, thereby shielding distal sites of vulnerabilities. Thus, the significance of a particular glycan in the V1 loop must be considered a function of loop length.

### Conclusions.

With the identification of helper lineages, there is increased interest in the role of ssNAbs that may facilitate breadth. We found that the overlapping ssNAb response may have constrained escape from the CAP177 bNAb response. This slow escape from the bNAb response, resulting in a prolonged exposure of the bNAb epitope, may have contributed to bNAb development. This finding is supported by slow escape observed for other bNAb lineages, including the V2 targeting donor CAP256 (28) and CD4 binding site targeting donor CH505 (3, 30). These results highlight a role for strain-specific responses in driving breadth, possibly by constraining certain bNAb escape pathways due to the overlapping polyclonal response.

## MATERIALS AND METHODS

### Study participant.

CAP177 is a participant in the Centre for the AIDS Programme of Research in South Africa (CAPRISA) 002 cohort, an acute infection study established in 2004 in Durban, South Africa (52).

### Next-generation sequence library preparation.

We used the primer ID method, which tags each RNA template with a unique identifier (33). Sequences were then grouped according to their ID tags, and a consensus sequence was derived for each group. The generation of consensus sequences largely removes PCR and sequencing errors, resulting in high-quality sequence data (53). Furthermore, as each RNA template has a unique sequence tag, it also controls for resampling, which occurs during PCR, thus enabling accurate quantification of viral variants. RNA extraction, cDNA synthesis, and amplification were carried out as described previously (33, 54), with the following modifications. A minimum of 5,000 HIV-1 RNA copies were isolated from longitudinal plasma, spanning 4 years of infection, using the QIAamp viral RNA kit (Qiagen, Germany). The cDNA synthesis primer was designed to bind to the C2 or C3 region of the envelope (HXB2 gp160 positions 658 to 683 and 1094 to 1118) and included a randomly assigned 9-mer tag (primer ID method) to uniquely label each cDNA molecule, followed by a universal primer binding site to allow out-nested PCR amplification of cDNA templates. First-round amplification primers were designed to amplify the V1/V2 or C2 to C3 regions of the envelope (HXB2 gp160 positions 332 to 358 and 721 to 749, respectively) and contained a template-specific binding region followed by a variable-length spacer of 0 to 3 randomly assigned bases to increase sample complexity. In addition, PCR primers contained 5′ overhangs, introducing binding sites for the Nextera XT indexing primers (Illumina, CA). The nested PCRs were carried out using the Nextera XT indexing kit. After indexing, samples were purified using the MinElute PCR purification kit (Qiagen, Germany), quantified using the Quant-iT PicoGreen double-stranded DNA assay (Invitrogen, CA), and pooled in equimolar concentrations. Size selection was performed on the pooled amplicons using the QIAquick gel purification kit (Qiagen, Germany) before submitting the final library for sequencing on an Illumina MiSeq (San Diego, CA) using 2- by 300-bp paired-end chemistry.

### Next-generation sequencing data processing and analysis.

Raw reads were processed using a local instance of Galaxy (55–57), housed within the University of Cape Town High-Performance Computing Core. Read quality was assessed using fastqc (http://www.bioinformatics.babraham.ac.uk/projects/fastqc). Short reads (<150 bp) and low-quality data were filtered out using the Filter FASTQ (version 1.0.0) tool (55), with a minimum quality of Q35 for 3′ base trimming. Forward and reverse reads were merged using PEAR (58). A custom python script was used to bin all reads containing an identical primer ID tag, to align the reads within each bin using MAFFT (59), and to produce a consensus sequence based on a majority rule. Sequences with a primer ID that was represented in fewer than three reads were discarded, along with those containing degenerate bases. The remaining sequences from each time point were aligned with MAFFT, Muscle, or RAMICS (60). Phylogenetic trees were drawn with FastTree v2.1 (61) and visualized using the python library ete3 (62). For the calculation of amino acid frequencies, V1-loop length, and glycosylation content, Shannon entropy (63) tests were performed using custom python scripts, and mapping of sequence entropy scores onto modeled structures of CAP177 was visualized using python scripts and the PyMOL molecular graphics system, version 1.8, Schrödinger, LLC. Figures were generated using Prism 6.07 (GraphPad, CA) or the python library Matplotlib (64). All custom scripts used in these analyses are available upon request.

### Structural modeling and glycosylation.

Crystal structures of the HIV-1 Envelope trimer and gp41 (PDB entries 4NCO, 4TVP, and 2B4C) were used as templates to create models from the CAP177 gp160 sequences, which was carried out using Modeler (65) and the UCSF Chimera package (66). The addition of high-mannose (Man-9) glycans to the modeled trimers was achieved using an in-house tool, developed by Oliver Grant and David Matten (unpublished data), which first explores the most populated rotamers of the Asn-GlcNAc linkage and then, if necessary, adapts the carbohydrate structure to its environment by iteratively rotating the interglycosidic linkages within normal bounds to resolve interglycan clashes (67). This automated process resolves potential steric clashes and increases the number of carbohydrates that can be attached to a glycoprotein substantially without the need for manual adjustment or minimization. Man-9 glycans were chosen to limit the computational complexity required, which increases exponentially when dealing with other oligomannose glycans which have multiple branching topologies.

### Single-genome amplification (SGA) and sequencing.

HIV-1 RNA was isolated from plasma using the Qiagen QIAamp viral RNA kit and reverse transcribed to cDNA using SuperScript III reverse transcriptase (Invitrogen, CA). The envelope genes were amplified from single-genome templates (68), and amplicons were directly sequenced using the ABI PRISM BigDye Terminator cycle sequencing ready reaction kit (Applied Biosystems, Foster City, CA) and resolved on an ABI 3100 automated genetic analyzer. The full-length *env* sequences were assembled and edited using Sequencher version 4.5 software (Genecodes, Ann Arbor, MI). Multiple sequence alignments were performed using Muscle (69) and edited with AliView (70).

### Cloning of *env* genes and site-directed mutagenesis.

Selected SGA amplicons were cloned into the expression vector pcDNA 3.1 (directional) (Invitrogen, CA) by reamplification of SGA first-round products using Fusion enzyme (Stratagene, CA) with the EnvM primer (71) and the directional primer EnvAStop (72). Cloned *env* genes were sequenced to confirm that they matched the sequenced amplicon exactly. SGA clones were mutated at positions 332 and 334 by site-directed mutagenesis using the Stratagene QuikChange II kit (Stratagene, CA) as described by the manufacturer. V1-loop mutants were constructed using overlap extension PCR with the primer EnvAdir, V1 loop-specific primers, and the EnvM primer. Mutations were confirmed by Sanger sequencing.

### Cell lines.

The TZM-bl cell line, engineered from CXCR4-positive HeLa cells to express CD4, CCR5, and a firefly luciferase reporter gene (under the control of the HIV-1 LTR), was obtained from the NIH Reagent Program. The HEK-293T cell line was obtained from George Shaw (University of Alabama, Birmingham, AL). Cells were cultured at 37°C, 5% CO_2_ in Dulbecco's modified Eagle's medium (DMEM) containing 10% heat-inactivated fetal bovine serum (Invitrogen, CA) with 50 μg/ml gentamicin (Sigma-Aldrich, MI) and disrupted at confluence by treatment with 0.25% trypsin in 1 mM EDTA (Sigma-Aldrich, MI).

### Generation of Envelope-pseudotyped viruses.

Pseudoviruses were generated by cotransfecting the *env* clones with the subtype B backbone vector pSG3Δenv (NIH AIDS Reagent Program) into 293T cells using FuGENE 6 transfection reagent (Roche, Switzerland). Cultures were incubated for 48 h to produce Env-pseudotyped viral stocks that were filtered through 0.45-μm filters and frozen in DMEM supplemented with 20% fetal bovine serum (FBS).

### Generation of IECs.

IECs were constructed by amplification of *env* from the *env* clones to introduce restriction enzyme sites and allow for cloning of these amplicons into a replication-competent pNL-LucR.T2A backbone vector (73). Infectious virus stocks were generated by transfecting 293T cells with the *env*-pnL-45LucR plasmids using PolyFect transfection reagent (Qiagen, Germany). IEC virus-containing supernatant was harvested 48 h after transfection and stored at −80°C.

### Neutralization assays.

Neutralization assays were performed in TZM-bl cells as previously described (37, 74). Neutralization is measured as a reduction in relative light units (RLUs) after a single round of pseudovirus infection in the presence of the monoclonal antibody or plasma sample of interest. Samples were serially diluted 1:3, and the ID_50_/50% inhibitory concentration (IC_50_) ratio was calculated as the dilution at which the infection was reduced by 50%. Viral escape was functionally confirmed as resulting in a >3-fold decrease in sensitivity to plasma neutralization.

### Single-cycle infectivity assay.

IEC viruses were serially diluted and added in triplicate to 10,000 TZM-bl cells (NIH AIDS Reagent Program) in the absence of DEAE-dextran. Luciferase activity was quantified after 48 h by adding Steadylite HTS (PerkinElmer, MA) and measuring luminescence with a Promega GloMax 96 luminometer. RLUs generated per volume of virus stock were calculated using all virus dilutions in the linear range of the assay (2,000 to 600,000 RLUs). The Gag p24 antigen content in each virus stock was quantified using the Allianz HIV-1 p24 antigen enzyme-linked immunosorbent assay (ELISA) kit (PerkinElmer, MA), and viral infectivity was subsequently calculated as the RLU per nanogram of p24 averaged over the dilutions and over two independent experiments.

### Ethics approval and consent.

The CAPRISA 002 acute infection study was reviewed and approved by the research ethics committees of the University of KwaZulu-Natal (E013/04), the University of Cape Town (025/2004), and the University of the Witwatersrand (MM040202). Participant CAP177 provided written informed consent.

### Accession number(s).

The deep-sequencing data sets generated during this study are available in the BioProject short read archive repository (accession numbers SRX2918056 to SRX2918105, available at http://www.ncbi.nlm.nih.gov/bioproject/390513). The CAP177 clonal sequences used have been deposited in the GenBank repository (accession numbers MF346585 to MF346598).
